# The Pathology of Leucocythæmia

**Published:** 1872-12

**Authors:** I. N. Danforth

**Affiliations:** Chicago


A MONTHLY RECORD OF

*Medicine, Surgery and the Collateral Sciences.*

EDITED BY J. ADAMS ALLEN, M.D., LL.D.; AND WALTER HAY, M.D.

VOL. XXIX. — DECEMBER, 1872. — NO. 12.

### Original Communications.

ARTICLE I.—*The Pathology of Leucocythæmia.* By I. N. DANFORTH, M.D., Chicago.

The true pathology of leucocythæmia cannot yet be regarded as positively or permanently settled. Yet the revelations of the dead room and of the microscope, coupled with a pretty uniform and definite succession of *ante mortem* events, are perhaps sufficient to warrant us in indulging in the luxury of forming an *opinion.*

The history of this singular disease is very interesting; for it is spiced by a wordy war between two of our modern medical giants, namely, Bennett and Virchow. It is worth while to glance briefly at this contest, and at the causes thereof, since it is closely related to other and not less important questions in pathology.

In the year 1841 a case of disease of the spleen was admitted into the Royal Infirmary, of Edinburgh, under the care of Dr. Cragie. Death took place on the 1st of April of the same year, in "consequence of purulent matter in the blood."* The late Dr. John Reid examined the case, and concurred with Dr. Cragie in

* Aitken's Practice, Vol. II, p. 109.

his conclusion. The latter inferred "that, by some means or other, purulent matter and lymph had been mixed with the blood, and, circulating with it, had given rise to the peculiar febrile and inflammatory symptoms which occurred during life, and to death in the manner in which it had taken place."* Seeing that the spleen had been for several weeks inflamed, was enormously enlarged, and was, moreover, the only organ diseased, he naturally and very logically inferred that the "purulent matter and lymph" could have come from no other source, and thus took the first step in the elucidation of the pathology of leucocythæmia. Recognizing a pathological condition which was "in some respects new," Dr. Cragie made careful notes of the case, but did not publish his observations till four years afterwards.

On the 27th of February, 1845, John Monteith, a slater, was admitted into the Edinburgh Royal Infirmary, under the care of Dr. Christison. He died on the 15th of March following, and four days after, the body was examined under the superintendence of Prof. Bennett.† The case was subsequently published by Bennett, in the *Edinburgh Medical and Surgical Journal*, as one of "hypertrophy of the spleen and liver, in which death took place from suppuration of the blood;" and although the most evident lesion during life was enlargement of the spleen, Dr. Bennett agrees with Dr. Cragie in thinking that the immediate cause of death was owing to the presence of purulent matter in the blood, notwithstanding the absence of any recent inflammation or collection of pus in the tissues, and that it produced the febrile symptoms."‡ In the same issue of the journal above referred to, Dr. Cragie's case was also published. Bennett, however, regarded his case as a particularly interesting one, since it demonstrated "the existence of *true pus*, formed universally within the vascular system, independent of any local purulent collection from which it could be derived;"§ while Cragie, as we have seen, believed the enlarged spleen was the true source of the so-called pus. In other words, Bennett believed that his case was one of true and general suppuration of the blood; that pus cells were formed in the *liquor sanguinis*, within the vessels, without any inflammation, or phlebitis, or other textural cause; while Cragie was of opinion that the

* Aitken, loc. cit.

† Bennett's Lectures, p. 815.

‡ Aitken, loc. cit.

§ Aitken, loc. cit.

inflamed and hypertrophied spleen was the real source of the white corpuscles.

Before alluding to the next historic phase of this subject I wish to call attention to one fact which seems to me to be of some importance, namely: the theory which Bennett then adopted concerning the origin of the superabundant white corpuscles was precisely in accordance with his present notions of spontaneous generation. He was then, as now, a pronounced advocate of spontaneous evolution, and it is by no means strange that he should have believed in the irresponsible origin of pus corpuscles. A plainer and broader-gauged putting of the case is this: the man who admits spontaneity at all, throws his compass overboard, and must not be blamed for guess-work navigation. While Bennett was toying with his bauble of spontaneity, Virchow was sinking a shaft in a mine which yielded *facts.*

About one month after the publication of Bennett's and Cragie's cases, Professor Virchow published his first article upon this disease in "Frorieps Notizen," his observations being based upon a case which had fallen under his notice *post mortem.* The same enormous increase of white blood corpuscles was observed; the same absence of inflammation of the blood vessels or elsewhere, and the same enlargement of the spleen. Spontaneous origin of pus cells in the blood was contrary to the pathological as well as the life-long biological doctrines of Virchow; hence, from very necessity, he fell back upon the enlarged spleen, regarding its increased as well as perverted functional power as the source whence the blood derived its great accession of white corpuscles. In 1845–6, several cases were recognized in England before death, and the knowledge of the morbid anatomy considerably increased. In 1847, Virchow published an exhaustive article upon this disease, conferring upon it the name of "leukæmia," and describing a new form, which consisted essentially in an enormous enlargement of the lymphatic glands, while the spleen was not materially altered. In 1851–2, Bennett reviewed the whole subject at length in a work entitled "Leucocythæmia, or White-all Blood," in which he abandoned his former view of suppuration of the blood, and adopted that of Virchow. Hereupon there ensued a lively war of words, mainly between Bennett and Virchow, but in which Kolliker also bore a part, concerning priority of discovery on Bennett's part, and correctness of pathological interpretation on the part of Virchow. The real facts in the case seem to me to be briefly this, judging from Aitken's account which appears to be severely impartial:

1st. Cragie first recognized the morbid condition of the blood, and partly interpreted its origin.

2nd. Bennett next recognized the morbid condition of the blood, and immediately, and in advance of any one else, published his observations, but totally misunderstood the pathology of the disease.

3rd. About one month thereafter, Virchow published his first article on white cell blood, and correctly explained its pathology.

So much for the history of leucocythæmia. Before discussing the nature of leucocythæmia, I wish to direct attention to certain cardinal facts which ought to be brought squarely to the front.

1st. The white corpuscles are *never* materially or permanently increased without some antecedent alteration of important organs.

2nd. The organs uniformly involved are the spleen, or the lymphatic glands; not unfrequently, the spleen AND lymphatic glands; in a small proportion of cases, the thyroid gland and supra-renal bodies have also been found changed.

3rd. The liver is generally more or less affected, especially in cases which are unusually chronic; but not until the disease has been for some time in progress; in other words, *not* as a *primary* or *necessary* element of the disease.

4th. The organs thus involved are always enlarged; generally to an immoderate extent—but sometimes death may even occur with only a medium degree of increase of bulk.

We have then this sequence of pathological events:

*a.* Enlargement of the spleen, lymphatic glands, thyroid gland and supra-renal bodies; or that group of organs which have long been known as "blood glands" or "blood-making glands."

*b.* Increase of the white blood corpuscles, and decrease of the red blood corpuscles.

*c.* Enlargement of the liver.

Hence the study of the pathology of leucocythæmia resolves itself into the study of the nature of the primary anatomical changes connected therewith.

By far the most frequent among these changes we find the enlargement of the spleen, and this constitutes the essence of that form of the disease which Virchow has named "splenæmia." Splenic leukæmia commences, according to Rindfleisch, with "a pure hyperæmia of the spleen,"* this produces tumefaction of the organ, and, after a longer or shorter time, is followed by a "considerable increase of volume of the pulp;"† in other words, a true and simple hyperplasia of the latter substance. At a later stage the malpighian bodies are crowded with blood, and also undergo a marked increase in size; this increase depends upon a rapid multiplication of colorless cells within these bodies, by the subdivision of previously existing cells, and also upon a corresponding increase of capillaries, so that each malpighian corpuscle has its functional capacity doubled or trebled. The connective tissue of the spleen is likewise increased, but only to the extent demanded by the increased size of the organ. To state the fact in the simplest manner, there is an *abnormal* development of *normal* splenic tissue—hence an *abnormal* performance of *normal* splenic *function.* This leads us directly to the question, "What is the function of the spleen?" In regard to this point, Rindfleisch observes: "The spleen has from all time been regarded as an important organ for the renewal of the blood; in our days, it has at one time been designated as the grave of the red corpuscles, at another as the birth place of the colorless; Kolliker has probably correctly ascribed to it both functions."‡ Without stopping to multiply quotations, it is sufficient to remark that the great majority of recent writers concur in believing that the spleen is directly concerned in the renewal of the white corpuscles, whatever may be its relation to the red globules. If this view be correct, the pathology of splenic leucocythæmia stands explained, so far as it can be at present; and if Kolliker's theory be accepted, it explains not only the increase of the white, but also the diminution of the red corpuscles as well. Both are to be attributed to the great increase of the functional capacity of the spleen, and this, in its turn, depends upon the immense growth of the proper tissue of the organ. Two interesting facts, which furnish an additional buttress to this view, are worth noting:

1st. Scherer has found in splenæmic blood, constituents foreign to normal blood, namely, lactic, acetic and formic acids, gelatine and

* Rindfleisch, p. 184.

† Op. cit., p. 185.

‡ Pathological Histology, p. 184.

hypoxanthin; but these same elements are also found in the pulp of the healthy spleen.

2nd. Barry, Murchison and Bennett have each met with a case of the disease in which *post mortem* examination revealed the presence of *two* spleens, one greatly hypertrophied, the other quite small; this—writes Dr. Barry—"would seem to indicate that in leucocythæmia there is a natural tendency to the formation of an exuberance of splenic tissue."*

That form of the disease which Virchow calls "lymphæmia" may be dismissed with few words, since it is precisely analogous to the splenic variety. We always find the lymphatic glands enlarged from a simple accretion of their normal tissue—in fact, a true hyperplasia. "If we examine fine teased out sections"—says Rindfleisch—"we find nothing that might not occur in a normal lymph-gland."†

In both forms of the disease, then, we have to deal with an immensely exaggerated physiological process—a process which we may properly characterize as pathologically physiological. Leucocythæmia is the product of an unnatural and uncalled-for growth of certain organs, just as the "big woman," and the "fat boy," who always intercept us as we emerge from the circus tent, are the product of an unnatural and uncalled-for growth of certain tissues; the one is as pathological and as physiological as the other. It is true that we sometimes find heteroplastic elements in the hyperplastic spleen, or lymphatic glands; but when this is the case, it is simply the introduction of a superadded element, which has no direct or necessary relation with the primary disease. Indeed, as a general thing, heteroplasia of the blood-forming glands, means the absence, rather than the presence, of leucocythæmia. It is true, also, that the liver is frequently enlarged in the course of this disease; but this is a secondary, not a primary process—the *consequence*, not the *cause* of leucocythæmia. The same thing has been observed with reference to the kidney by Virchow; in one case examined by him the liver was crowded with white corpuscles, and this infiltration followed the ramifications of the portal vein, and the cells were evidently conveyed there by that vessel. In the same instance, the kidneys were also infiltrated with white corpuscles; and the influence is fair and logical that these organs were vainly

* Beale's Archives, Vol. II, p. 5.

† Op. cit., p. 186.

trying to rid the blood of its superabundance of leucocytes. It is all but needless to say that these facts have no relation, direct or indirect, with the disease *per se*.

In relation to the underlying cause of leucocythæmia, we know precisely *nothing*. We can trace it to modified, or, more correctly, exaggerated cell action, but there, for the present, we must stop.

Virchow speaks of a "lymphatic diathesis," but this conveys no real meaning. We might as well talk of a "fighting diathesis" in connection with a quarrelsome man, or a "loafing diathesis" in connection with a lazy man. Leucocythæmia sometimes follows inflammation of the spleen or of the lymphatic glands; but why it follows inflammatory action in some cases and not in others, we are unable to say. In the study of this disease, as of all others, at a certain point in our investigations we find ourselves confronted by ultimate facts, which are, in the present state of our knowledge, inexplicable. In this case, therefore, as in a legion of others, we must be content to trust the developments of future years.

In conclusion, I wish to ask attention to one more point, which, for me at least, possesses a novel interest; namely, the structural analogy existing between leucocythæmic and reptilian blood. The cardinal point of difference between mammalian and reptilian blood, so far as the microscope has determined, consists in this: that the former contains a large proportion of non-nucleated corpuscles or disks, and an exceedingly small proportion of nucleated cells, while the corpuscles of the latter are always nucleated. Hence the blood of patients laboring under leucocythæmia approaches, in its formed elements, the structure of reptilian blood. Add to this the great increase in the so-called "lymphadenoid" tissues, the slowness of the heart's action, the reduction of the temperature of the body, and the sluggish condition of the nervous system, all of which are peculiarities of reptilian life, and we have a singular chain of facts which are not altogether devoid of interest.

